# Band Structure, Phonon Spectrum and Thermoelectric Properties of Ag_3_CuS_2_

**DOI:** 10.3390/ma16031130

**Published:** 2023-01-28

**Authors:** Dmitry Pshenay-Severin, Satya Narayan Guin, Petr Konstantinov, Sergey Novikov, Ekashmi Rathore, Kanishka Biswas, Alexander Burkov

**Affiliations:** 1 Ioffe Institute, St Petersburg 194021, Russia; 2 Jawaharlal Nehru Centre for Advanced Scientific Research, Bengaluru 560064, India

**Keywords:** jalpaite, thermoelectric properties, Seebeck coefficient, electric conductivity, thermal conductivity

## Abstract

Sulfides and selenides of copper and silver have been intensively studied, particularly as potentially efficient thermoelectrics. Ag${}_{3}$CuS${}_{2}$ (jalpaite) is a related material. However very little is known about its physical properties. It has been found that the compound undergoes several structural phase transitions, having the tetrahedral structural modification I4${}_{1}$/amd at room temperature. In this work, its band structure, phonon spectrum and thermoelectric properties were studied theoretically and experimentally. Seebeck coefficient, electrical conductivity and thermal conductivity were measured in a broad temperature range from room temperature to 600 K. These are the first experimental data on transport properties of jalpaite. Ab initio calculations of the band structure and Seebeck coefficient were carried out taking into account energy dependence of the relaxation time typical for the scattering of charge carriers by phonons. The results of the calculations qualitatively agree with the experiment and yield large values of the Seebeck coefficient characteristic for lightly doped semiconductor. The influence of intrinsic defects (vacancies) on the transport properties was studied. It was shown that the formation of silver vacancies is the most probable and leads to an increase of hole concentration. Using the temperature dependent effective potential method, the phonon spectrum and thermal conductivity at room temperature were calculated. The measurements yield low lattice thermal conductivity value of 0.5 W/(m K) at 300 K, which is associated with the complex crystal structure of the material. The calculated room temperature values of the lattice thermal conductivity were also small (0.14–0.2 W/(m K)).

## 1. Introduction

In the present work, the band structure, Seebeck coefficient and lattice properties of copper and silver sulfide—Ag${}_{3}$CuS${}_{2}$ (mineral jalpaite) were studied. Sulfides and selenides of copper and silver were intensively studied, particularly in thermoelectricity. Interest in the family of these materials arose in connection with the concept of “phonon glass, electron crystal” (PGEC), put forward by Slack [1]. In copper selenide Cu${}_{2}$Se rather high values of thermoelectric figure of merit $ZT=S^{2}\sigma /\kappa$ of up to 0.4–1.6 in the temperature range 500–1000 K were obtained [2,3] (here, σ and κ are electrical and thermal conductivities, *S* is the Seebeck coefficient and *T* is the absolute temperature). In this temperature range, copper selenide is in a superionic cubic β phase with the antifluorite structure (transition temperature 413 K). The increased copper ion mobility leads to a strong disorder in the cationic sublattice and to a strong scattering of phonons, resulting in the deacrease of the thermal conductivity down to 0.5 W/(m K), which is one of the reasons for the high thermoelectric figure of merit of the material. This led to the extension of the PGEC concept and the emergence of a new paradigm—”phonon liquid, electron crystal” (PLEC) [2]. In addition to thermoelectric applications, copper selenide was studied as a material for solar cells [4,5]. The study of the electronic spectrum showed that it is a direct band gap semiconductor with a band gap of 1.1–1.73 eV [5,6,7].

High mobility of copper ions in the high-temperature phase was also observed in more complex silver and copper sulfides [8,9]. According to the literature data, Ag${}_{3}$CuS${}_{2}$ undergoes several structural phase transitions [10,11]. At room temperature, it has a tetragonal structure. With increasing temperature the material undergoes a transition to a body-centered (at 387 K) and then to a face-centered cubic phase (at 549 K). According to X-ray data, a strong disorder was observed in Cu-sublattice of both cubic phases, which led authors to the conclusion that the phases are superionic. At room temperature, Ag${}_{3}$CuS${}_{2}$ is a direct-gap semiconductor with band extrema at the Γ point of the Brillouin zone with a band gap of 1.05 eV [12], therefore, besides thermoelectric, it was also considered for photovoltaic applications [13,14].

**Figure 1:**
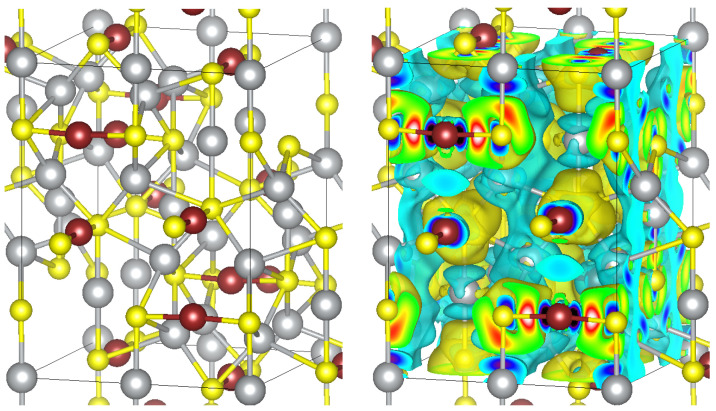
Body-centered tetragonal unit cell of Ag${}_{3}$CuS${}_{2}$ in jalpaite I4${}_{1}$/amd structure (**left** panel). Ag, Cu and S atoms are depicted using gray, brown and yellow spheres, respectively. Redistribution of electron density (**right** panel). The yellow contours correspond to an increase, and the cyan contours correspond to a decrease in the electron density compared to the electron density of atoms placed at the same positions.

At room temperature, Ag${}_{3}$CuS${}_{2}$ exists in a tetragonal modification (space group I4${}_{1}$/amd, No.141). There are 4 formula units (24 atoms) in the unit cell, but often the structure is represented using a non-elementary body-centered tetragonal cell (8 formula units, 48 atoms; see the Figure 1, left panel). Ag atoms occupy two non-equivalent positions [10,11]: 8c positions (0, 0, 0) for Ag1 and 16g positions (−0.3127, −0.0627, 0.875) for Ag2. The environment of silver atoms can be represented as a distorted octahedra (2 + 4) for Ag1, in which 2 of the surrounding sulfur atoms are at a distance of 2.530 Å, and the remaining 4 are at a distance of 3.072 Å. For silver atoms in the Ag2 position, the environment is described as a distorted tetrahedral (2 + 2) with two closer sulfur atoms at a distance of 2.552 Å and two farther apart at a distance of 2.957 Å. The Shannon radius for sulfur is 1.7 Å; for silver in the 2-, 4-, and 6-coordination configurations, they are 0.81, 1.14, and 1.29 Å, respectively. The smallest of the distances to sulfur atoms in both silver configurations are in good agreement with the sum of the Shannon radii for sulfur and silver in the two-coordination configuration −2.51 Å. The sum of the Shannon radii for the 4- and 6-coordination environments of silver and sulfur are 2.84 and 2.99 Å, that are closer to the distances to farther S atoms in these configurations. The copper atoms are in positions 8e (0, 0.25, 0.5319). Their closest neighbors are 2 sulfur atoms located almost on the same straight line at a distance of 2.1822 Å [11]. For comparison, the sum of the Shannon radii of sulfur and copper in the 2-coordination configuration (0.6 Å) for this case is 2.3 Å. Below the room temperature, at 250 K, Ag${}_{3}$CuS${}_{2}$ transforms into another tetragonal phase I4${}_{1}$/a of similar atomic coordination but with reduced symmetry of interatomic distances.

To our knowledge, there is no information available in literature on electronic and thermal transport properties of Ag${}_{3}$CuS${}_{2}$.

## 2. Sample Preparation and Characterisation

Samples of Ag${}_{3}$CuS${}_{2}$ were prepared by melting of appropriate amounts of the constituting elements. The elemental silver (Ag, 99.999%, metal basis, Alfa Aesar), elemental copper (Cu, 99.9999%, metal basis, Alfa Aesar), and elemental sulfur (S, 99.999%, Alfa Aesar) were mixed in appropriate ratios in a quartz tube. The tubes were sealed under high vacuum (∼10${}^{-5}$ Torr) and slowly heated to 773 K over 12 h, then heated to 1273 K in 5 h, soaked for 24 h, and subsequently slow cooled to room temperature. The structure of the synthesized compounds was characterized by X-ray powder diffraction.

Thermoelectric properties - Seebeck coefficient, *S*, electrical resistivity, ρ, and thermal conductivity, κ, - were measured using two home-made setups. The thermal conductivity was measured by steady-state classic procedure with setup, described in Ref. [15]. The setup is designed to make simultaneous measurement of thermal conductivity, electrical resistivity and Seebeck coefficient. However, the resistance of the Ag${}_{3}$CuS${}_{2}$ samples was too high for simultaneous resistivity and Seebeck coefficient measurements. Therefore, using this setup only the thermal conductivity was measured at temperatures from 100 K to 600 K. The resistivity and Seebeck coefficients were measured with another setup with higher input impedance and therefore capable to measure samples with higher resistance [16,17]. The standard uncertainty of the thermal conductivity measurements is below ±10%. This uncertainty is mainly related to the uncertainty of determination of the heat energy density passing through sample crossection. Usually the samples have a regular, high quality shape, therefore the sample cross-section determination error is small. However in the present case the cylindrical samples of Ag${}_{3}$CuS${}_{2}$ have porous imperfections, that are difficult to take into account accurately when measuring the sample cross-section. Therefore we rise the error bar for the thermal conductivity in the present case to about 15%. The electrical resistivity 4-point DC measurement uncertainty is mainly determined by the shape factor, i.e., uncertainty of determination of the sample cross-section, and in the present case we estimate it as ±5%. The Seebeck coefficient measurement uncertainty was estimated as ±(5% + 0.5 μV/K) [16]. However, to comply with this uncertainty, the sample resistance should not exceed the value of order 100 kOhm.

## 3. Experimental Results

**Figure 2:**
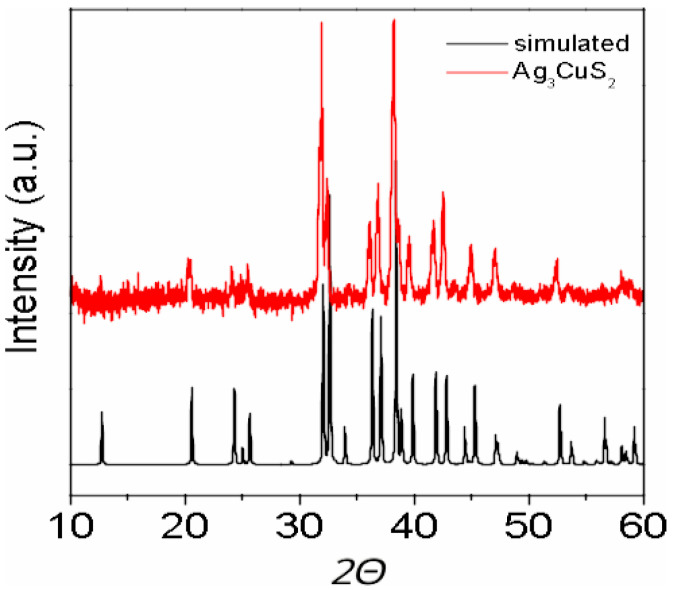
X-ray experimental diffraction pattern of Ag${}_{3}$CuS${}_{2}$ and simulated X-ray diffraction of I4${}_{1}$/amd structure.

The powder XRD results, shown in Figure 2, confirm the tetragonal crystal structure without significant amount of impurity phases.

**Figure 3:**
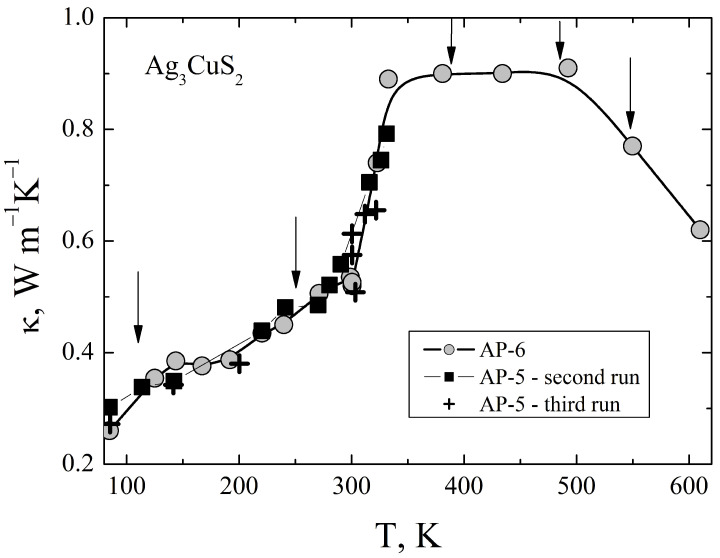
Thermal conductivity of Ag${}_{3}$CuS${}_{2}$. The arrows indicate temperatures of the structural phase transitions according to Ref. [11].

The thermal conductivity was measured from 100 K to 600 K. To cover this temperature range we use two setups. In both cases the same steady-state method of the measurements was used, however at lower temperatures, from 100 K to 350 K–380 K, the radiation heat losses from the sample are comparatively small and are taken into account by introducing calibrated corrections [17], whereas at higher temperatures special sample holder room with active thermal guard shielding is used to suppress the radiation heat losses [15]. The measurements results are presented in Figure 3.

The thermal conductivity of the compound is very low and shows an unusual temperature dependence. It attains the maximum value of 0.7 WK${}^{-1}$m${}^{-1}$ above 300 K, is almost independent of temperature up to 500 K, and decreases linearly at 500 K to 600 K. Below 300 K the thermal conductivity is roughly a linear function of temperature. At 100 K it has value of only 0.3 WK${}^{-1}$m${}^{-1}$. The sample resistivity is typical for undoped semiconductors and is very high, therefore the electronic contribution to the thermal conductivity is negligible. As it was mentioned in Introduction, Ag${}_{3}$CuS${}_{2}$ undergoes a series of structural phase transitions: P−based <–110 K–> $I4_{1}/a$ <–250 K–> $I4_{1}/amd$ <–387 K–> Im3m <–483 K–> Im3M+Fm3m <–549 K–>Fm3m [11]. There is a clear correspondence between these phase transition temperatures and peculiarities on the temperature dependence of the thermal conductivity at 483 K, and possibly at 110 K and 250 K. However, the sharp increase of the thermal conductivity at 300 K apparently does not correspond to a phase transition. On the other hand, the phase transition $I4_{1}/amd$ <—> Im3m at 387 K has no clear signature in the temperature dependence of the thermal conductivity. According to Ref. [11] this structural transition is accompanied by appearance of superionic phase with large structural disorder. Therefore one can expect a decrease of the lattice thermal conductivity at this temperature, which, however is not observed in our results. More detailed investigations of the thermal conductivity with thermal cycling across the transition at 387 K are necessary to resolve this apparent contradiction.

**Figure 4:**
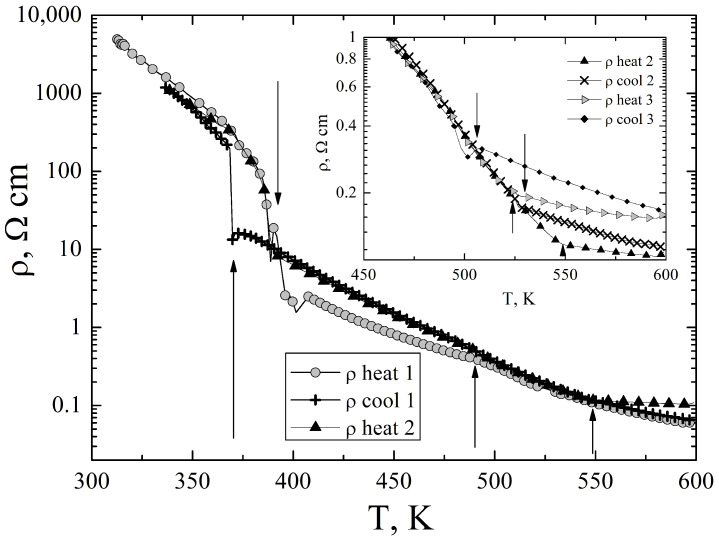
Electrical resistivity of Ag${}_{3}$CuS${}_{2}$. The arrows indicate temperatures at which the resistivity has peculiarities, these temperatures well correlate with the structural phase transitions according to Ref. [11]. The inset shows the evolution of the peculiarity near to 500 K with thermal cycling. The upward pointing arrows indicate the on-heating temperature of the transition, while the downward pointing arrows show the on-cooling transition temperature according to the resistivity data.

The electrical resistivity of Ag${}_{3}$CuS${}_{2}$ is shown in the Figure 4.

**Figure 5:**
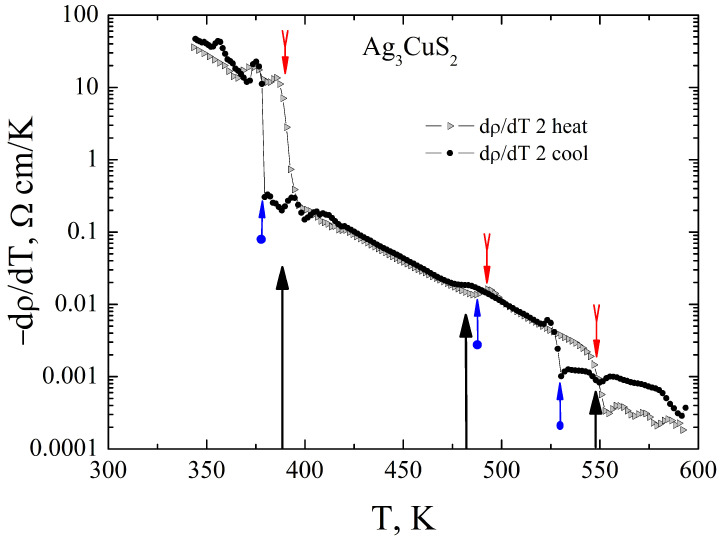
Temperature derivative of the resistivity of Ag${}_{3}$CuS${}_{2}$ on the second heating-cooling cycle. The large arrows indicate temperatures of the structural phase transitions according to Ref. [11]. The small downward pointing arrows indicate the on-heating temperature of the transition, while the upward pointing arrows show the on-cooling transition temperature according to the resistivity data.

The resistivity was measured in dynamical temperature regime, i.e., with controlled continuous temperature variation during the measurements. The temperature variation rate in these measurements was about 5 K/min. The resistivity is large and has typical for undoped semiconductors temperature variation. The Figure 4 presents the resistivity temperature dependence from 300 K to 600 K, measured in three heating-cooling cycles of one Ag${}_{3}$CuS${}_{2}$ sample. On heating above 370 K a sharp drop of the resistivity is observed with pronounced temperature hysteresis on cooling. This behaviour of the resistivity is typical for a first-order phase transition, the temperature coincides with the temperature of the $I4_{1}/amd$ <—> Im3m transformation [11]. The resistivity temperature dependence around this temperature is well reproducible on thermal cycling. The transition temperature, according to the resistivity data is 390 K±2 K on heating, and 375 K±10 K on cooling. There are another, less pronounced, peculiarities on the resistivity temperature dependence: change of the slope of lnρ vs *T* dependence near to 480 K and 550 K. These peculiarities are more clearly visible on the temperature dependence of the resistivity temperature derivative dρ(T)/dT, Figure 5. The temperatures of these peculiarities roughly correspond to the temperatures of the phase transitions Im3m <–483 K–> Im3M+Fm3m and Im3M+Fm3m <–549 K–>Fm3m [11]. However, as it is demonstrated in the inset to the Figure 4, the temperatures and even the magnitude of the peculiarities are dependent on the thermal history of the samples.

**Figure 6:**
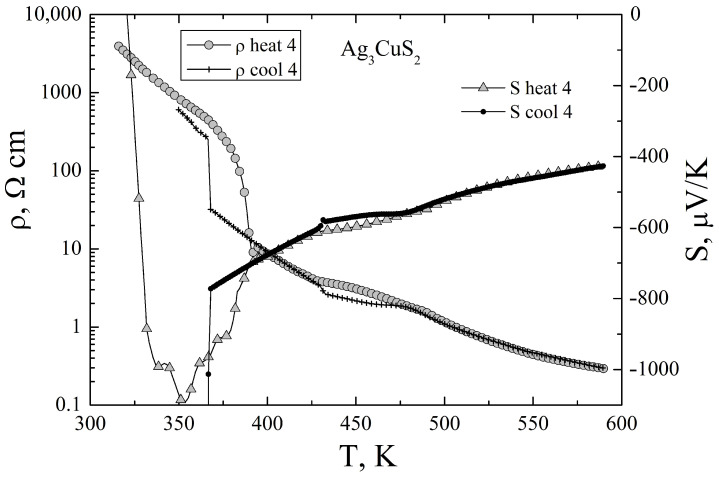
Temperature dependencies of the Seebeck coefficient and of the resistivity of Ag${}_{3}$CuS${}_{2}$ sample on the fourth heating-cooling cycle. The inset shows the thermoelectric power factor $S^{2}/\rho$.

The Seebeck coefficient was the most difficult to measure because of the high resistivity of the samples. Therefore sufficiently reliable data on the Seebeck coefficient were obtained only at temperatures above $I4_{1}/amd$ <—> Im3m transition. The results, together with simultaneously measured resistivity, are presented in Figure 6.

The Seebeck coefficient at high temperatures is large in magnitude and has negative sign, the magnitude and the temperature variation are consistent with n-type semiconductor in intrinsic conductivity regime. Immediately below $I4_{1}/amd$ <—> Im3m transition the Seebeck coefficient is negative. However we can not reliably measure its temperature variation and even the sign with further decrease of temperature.

In spite of the large Seebeck coefficient, the thermoelectric power factor $S^{2}/\rho$ of the compound, shown in the inset of the Figure 6, is very small with maximum value of about 0.6 μW/cm K${}^{2}$ at 600 K. This is due to the large electrical resistivity of the undoped compound. Further studies of the compouns are neccesary to find suitable ways for charge carrier optimisation.

**Figure 7:**
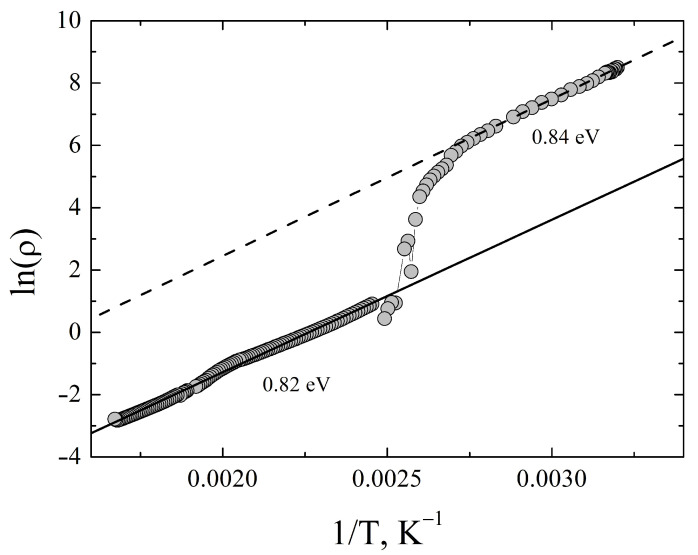
The dependence of the lnρ(T) vs 1/T for Ag${}_{3}$CuS${}_{2}$ sample.

Using the resistivity temperature dependence we estimate the band gap of the compound, Figure 7.

The estimate gives roughly the same band gap of ≈0.8 eV for the room-temperature phase $I4_{1}/amd$ and for all high-temperature modifications. This value of the band gap is considerably higher than theoretical one obtained using gradient-corrected local density functional approximation, discussed below. However, it is in quite good agreement with the value of 1.05 eV, obtained from optical reflectance data and hybrid functional calculations [12].

## 4. Ab Initio Calculation of Band Structure and Transport Properties

Ab initio calculations of lattice parameters and band structure were performed using density functional theory as implemented in VASP program [18,19]. We used generalized gradient PBE approximation for the density functional, plane wave energy cutoff of 400 eV and Monkhorst-Pack grid of 4×4×4. The equilibrium lattice parameters were obtained through the calculation of equation of states with the unit cell shape and atomic force relaxation better than 1 meV/Å. The obtained parameters a= 8.8974 Å, c= 11.6566 Å can be compared with the experimental values a= 8.6476 Å, c= 11.7883 Å from Ref. [11] and a= 8.6705 Å, c= 11.7573 Å from Ref. [10]. The averaged deviation from experimental values for *a* and *c* are 2.8% and −1.0%, respectively. The calculations by Savory [12] gave larger deviations in the case of PBEsol density functional (−4.9% and 3.9%), but the use of hybrid functional allowed them to improve the agreement with experiment, e.g., in HSE06 approximation the deviation was 2.2% and 0.1% [12]. Here, we used the values obtained in PBE approximation that provided a balance of accuracy and computational efficiency. The atomic positions, obtained in this approximation, are given in the Table 1 and demonstrate good agreement with the experiment [11].

To investigate bonding character of Ag${}_{3}$CuS${}_{2}$, we calculated electron density. The redistribution of the electron density in the crystal compared to the density in atoms at the same positions is shown in the Figure 1 (right panel). One can see an increase in the electron density around the sulfur atoms and a decrease around silver and copper. Numerically, this redistribution can be characterized by the Bader ionic charges, which amounted to 0.38 (Ag1), 0.32 (Ag2), 0.3 (Cu), and −0.67 (S). For comparison, in PbTe the effective ion charges are ±0.64, and in NaCl they are ±0.86. Considering that there are 4 metal cations per 2 sulfur ions, the degree of ionicity is apparently close to that of PbTe. Another way to estimate the degree of ionicity *f* of a compound is using the electronegativity difference ΔX according to the formula $f=1-\exp(-0.67\Delta X^{2})$ [20]. Using the electronegativity values from [20]$X_{\mathrm{Pb}}$ = 2.62 and $X_{\mathrm{Te}}$ = 3.14, one can get f=17% for PbTe. Estimations using Pauling’s electronegativities gave ionicity for PbTe about 20% [21]. For silver and copper sulfide, taking into account the close electronegativity values of the metals $X_{\mathrm{Ag}}$ = 2.88 and $X_{\mathrm{Cu}}$ = 2.86, and also $X_{S}$ = 3.44, a similar value f=19% was obtained. Thus, the bonding character in Ag${}_{3}$CuS${}_{2}$ is of covalent-ionic type with a degree of ionicity of about 19%.

Recently, another approach to the estimation of bonding character was proposed in [22]. It is based on a charge-transfer index *c*, calculated from Bader charges $Q_{i}$ and nominal oxidation state $Q_{ox,i}$ of the *i*-th ion: $c=\left(1/N\right)\sum_{i=1}^{N}Q_{i}/Q_{ox,i}$, where *N* is a total number of atoms. Using obtained Bader charges and nominal oxidation states of Ag${}^{1+}$, Cu${}^{1+}$ and S${}^{2-}$, one can obtain charge-transfer index for jalpaite c=0.33. For comparison, the calculations for PbTe gave c=0.32. According to [22], small values of *c* correspond to covalent bonding, *c* = 0.3–0.6 is typical for polar III-V compounds and nitrides, and the values close to unity correspond to ionic bonding. That is the use of charge-transfer index leads to qualitatively similar conclusion of mainly covalent type of bonding in jalpaite with a degree of ionicity close to that in PbTe.

**Figure 8:**
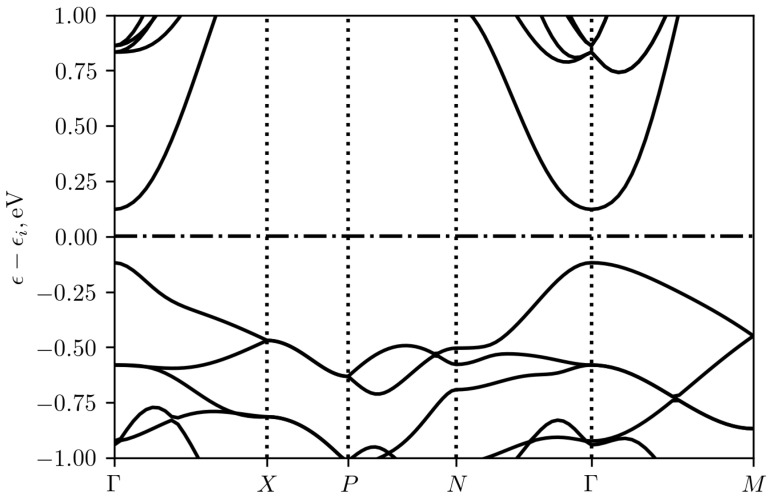
Band structure of Ag${}_{3}$CuS${}_{2}$ (I4${}_{1}$/amd), calculated using generalized gradient PBE approximation. Energy is counted from the middle of the band gap $\epsilon_{i}$. Special points in the Brillouin zone are denoted according to [23].

Figure 8 shows the results of band structure calculations for Ag${}_{3}$CuS${}_{2}$ (I4${}_{1}$/amd) in the PBE approximation. On the whole, they agree with those given in [12] except much smaller band gap $\epsilon_{g}=$ 0.24 eV. This is a well known deficiency of the PBE approximation which usually underestimates the band gaps. The larger value of $\epsilon_{g}=1.05$ eV was obtained using HSE06 hybrid functional by Savory [12] and was confirmed in our calculations of the band energies at the Γ point in the same approximation. The value of 1.05 eV was also obtained by Savory [12] experimentally in optical reflectance measurements.

**Figure 9:**
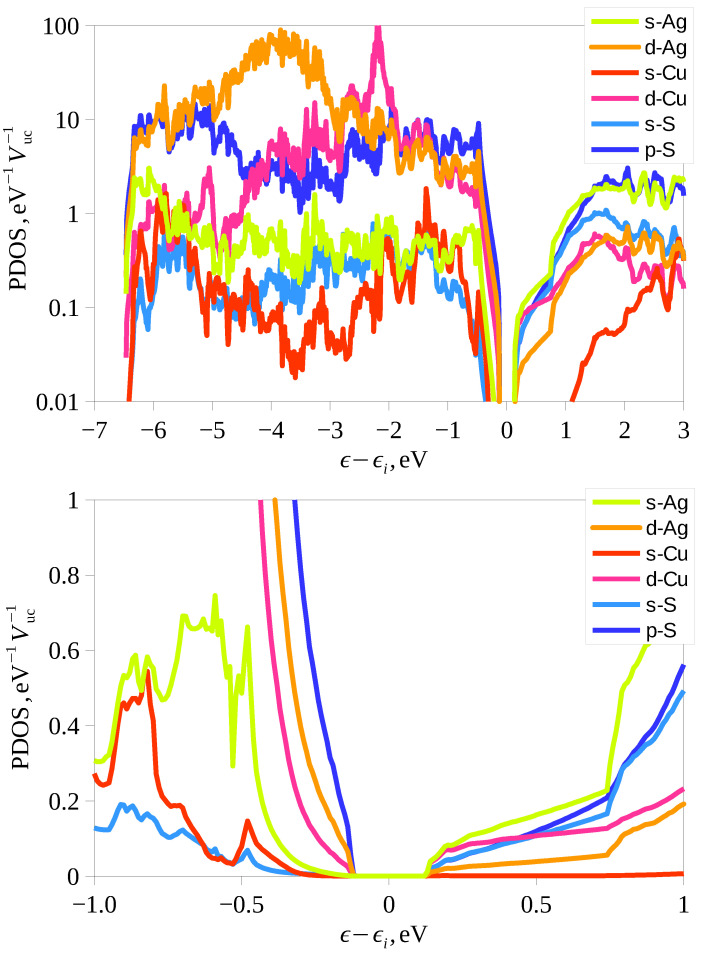
Atomic state projected density of electron states (PDOS) in Ag${}_{3}$CuS${}_{2}$ (I4${}_{1}$/amd). Upper panel shows PDOS in logarithmic scale in the wide energy range, while lower panel shows PDOS around the band gap. Energy is counted from the middle of the band gap $\epsilon_{i}$.

Ag${}_{3}$CuS${}_{2}$ turns out to be a direct band gap semiconductor with band extrema at the Γ point. Figure 9 shows the energy dependencies of the atomic orbitals projected density of states. It can be seen that the main contribution near the top of the valence band comes from the p-S, d-Ag, and d-Cu states with a small addition of the s-Ag contribution. In the depth of the valence band, the contribution of the d states of cations and the p states of sulfur dominates. The s states of sulfur form mainly a narrow band approximately 13 eV below the considered valence band (not shown in the Figure 9). The states near the bottom of the conduction band are formed by s-Ag, d-Cu, p-S, s-S, d-Ag in the order of decreasing contribution.

**Figure 10:**
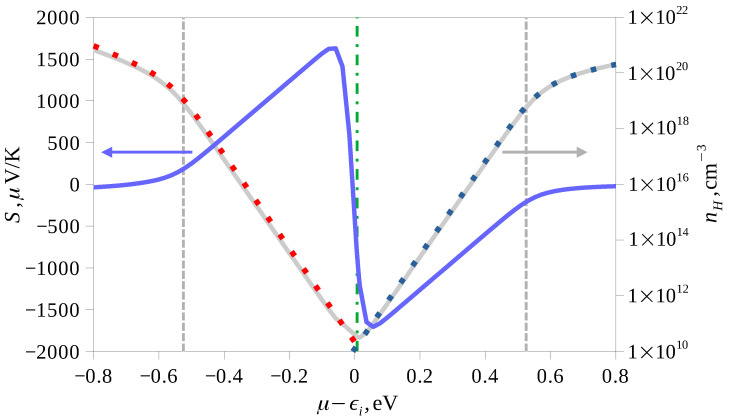
The dependence of the Seebeck coefficient (blue curve, left axis) and the Hall concentration (gray curve, right axis) on the chemical potential at 300 K. The vertical gray dashed lines show the boundaries of the band gap, and the green dash-dotted line shows the position of the chemical potential at equal concentrations of electrons and holes. Red (blue) dots on the Hall concentration plot show the concentrations of holes (electrons) separately. The chemical potential is counted from the middle of the band gap $\epsilon_{i}$.

The Seebeck coefficient and the Hall concentration were calculated using band structure interpolation on 32×32×32 k-point grid in BoltzTraP [24] program. For calculation of the transport coefficients, the energies of the conduction band states were shifted upward by 0.8 eV to correct $\epsilon_{g}$ using the so-called scissors operator. For the relaxation time, the energy dependence τ(ϵ)∼1/DOS(ϵ) was used, which is typical for the scattering of charge carriers on acoustical phonons. The results of calculations at room temperature are shown in the Figure 10. The position of the chemical potential at 300 K for intrinsic conduction, when the concentrations of electrons and holes are the same, is slightly shifted from the middle of the band gap $\epsilon_{i}$ to the bottom of the conduction band (by about 7 meV). This agrees with the band structure plot and the density of states: the latter is higher near the top of the valence band than near the bottom of the conduction band. Estimates of the effective masses of the density of states near the band extrema also gave larger values for holes $m_{p}=0.64m_{0}$ than for electrons $m_{n}=0.49m_{0}$. If the proportionality constant in τ(ϵ) dependence is the same for electrons and holes, the mobility ratio $u_{n}/u_{p}$ for non-degenerate statistics turns out to be 2.4. The Seebeck coefficient in the case of intrinsic conduction turns out to be negative and equal to about −800 μV/K at room temperature. The maximum values of Seebeck coefficient depending on the concentration are in the range of ±1700 μV/K. By the order of magnitude, it agrees with the measured values of Seebeck coefficient above room temperature, which exceeds −1000 μV/K. At the same time, the experimental resistivity is greater than 1 kOhm·cm, i.e., apparently, the carrier concentration in the sample corresponds to the intrinsic conduction, which at room temperature, taking into account the magnitude of the band gap, should be small. The experimental data indicate a possible change of the Seebeck coefficient sign from positive to negative near room temperature that should be connected with the phase transition. A similar behavior was observed in undoped AgCuS [25,26] samples, in which a double p-n-p conductivity sign reversal associated with two phase transitions at 361 K and 439 K was found. In that case, the range of Seebeck coefficient variation exceeded 1700 μV/K. Calculations showed [25] that the change in sign of the Seebeck coefficient in AgCuS was due to passing of the system through an intermediate semimetallic state in the course of the phase transition at 361 K, when the chemical potential was shifted from the top of the valence band to the bottom of the conduction band and back.

To optimize Ag${}_{3}$CuS${}_{2}$ for thermoelectric applications, it is necessary to find methods of its alloying. As a first step, we performed supercell calculations for a crystal in the presence of native point defects—copper, silver, and sulfur vacancies. We used a supercell with 96 atoms containing 16 formula units. In the supercell, one of the selected atoms was removed, and the positions of atoms in the presence of a defect were optimized up to residual interatomic forces less than 0.01 eV/ Å while the cell shape and the volume were kept unchanged to better model the limit of low impurity concentration [27]. For each of the considered defects, the density of electronic states was calculated on a 9×9×9 k-point grid and the anticipated type of doping was determined from the shift of electronic chemical potential. The defect formation energies were calculated following the method described in Ref. [27]. The obtained formation energies appeared to be negative: $\epsilon_{f}\left(V_{\mathrm{Ag}}\right)=-0.58$ eV, $\epsilon_{f}\left(V_{\mathrm{Cu}}\right)=-0.43$ eV and $\epsilon_{f}\left(V_{S}\right)=-0.53$ eV. This is connected to the fact that considered structural modification of jalpaite occurs at room temperature, and the total energy calculations were carried out for zero temperature. Thus, obtained results requires further corrections due to lattice contribution and were used here only for qualitative conclusions on the relative probability of defects formation.

**Figure 11:**
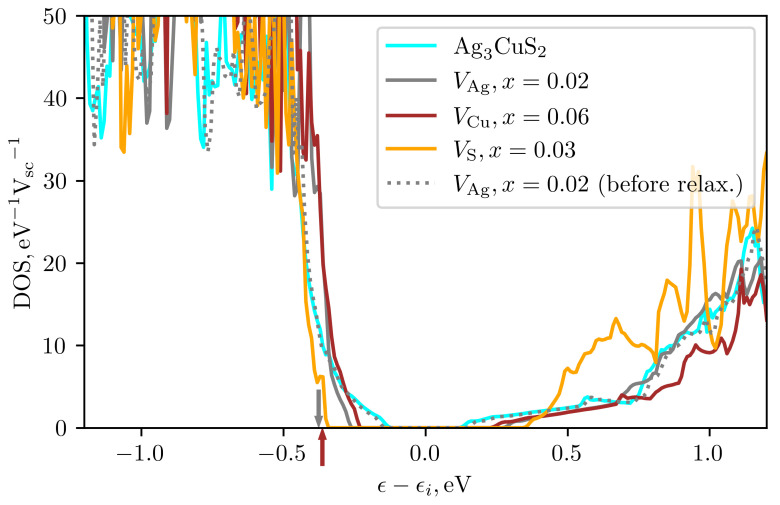
The density of states around the band gap of pure Ag${}_{3}$CuS${}_{2}$ and in the presence of Ag, Cu, and S vacancies with the atomic fraction *x*. The arrows show the positions of Fermi energy for Ag and Cu vacancies. Gray dotted curve shows DOS in the presence of Ag vacancy before atomic relaxation. Energy is counted from the middle of the band gap $\epsilon_{i}$.

The calculations showed that the formation of a silver vacancy is most probable, followed by that of sulfur and copper. Figure 11 shows the energy dependences of the density of states near the band gap in comparison with pure Ag${}_{3}$CuS${}_{2}$. Calculations showed that in the presence of copper or silver vacancies the chemical potential is shifted down into the valence band, which suggests that these defects should lead to the p-type doping. In the case of sulfur vacancies the valence band remains completely filled and doping does not occur. In all considered cases, simultaneously with doping, the band gap increases, which is especially strong in the presence of sulfur vacancies, which is obviously related to the large contribution of the p states of sulfur to the density of states near the band gap boundaries. The comparison of DOS before and after ionic relaxation for the case of Ag vacancies (see gray dotted and solid curves in the Figure 11) showed that an increase in the band gap occurs mainly during the process of atomic relaxation. In all considered cases, the density of states in the valence band in the presence of vacancies increases with the hole energy steeper than in pure Ag${}_{3}$CuS${}_{2}$. Thus, the formation of vacancies should be accompanied by an increase of hole effective mass. The presence of cation vacancies and the effect p-type doping was also experimentally observed in the related compound AgCuS [26].

## 5. Ab Initio Calculation of Phonon Spectrum and Lattice Thermal Conductivity

**Figure 12:**
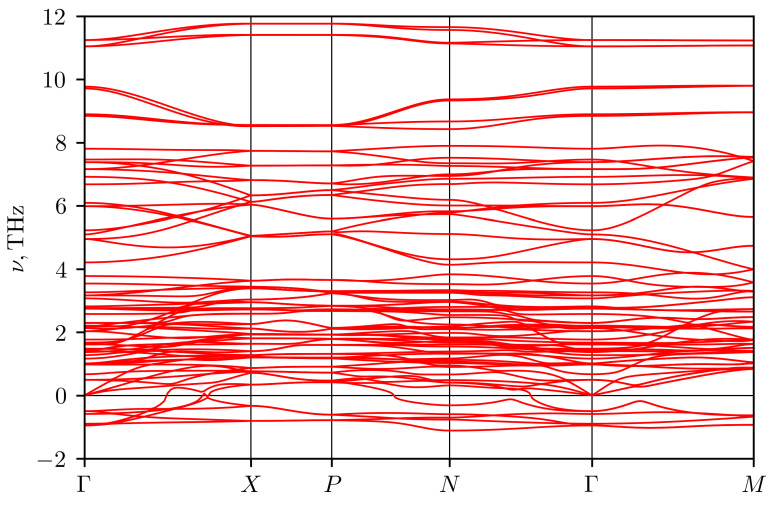
Phonon spectrum of Ag${}_{3}$CuS${}_{2}$ (I4${}_{1}$/amd), calculated at zero temperature, using equilibrium lattice parameters and PBEsol density functional approximation [28].

As was mentioned above, the crystal structure at zero temperature differs from the structure at room temperature. The available data on the phonon spectrum demonstrate imaginary phonon frequencies (see the Figure 12), indicating an instability of the tetragonal modification at zero temperature [28]. Therefore, in the present work, we took into account the stabilization of phonon modes due to anharmonicity. The calculation was carried out using the temperature dependent effective potential approach (TDEP) [29,30]. In this method, the potential energy of atoms in a crystal is expanded into a series in terms of atomic displacements with expansion coefficients that depend on temperature. To determine these coefficients, we performed molecular dynamics modeling of the crystal at room temperature. The effective force constants of the 2nd and 3rd orders were determined using the least squares method from the data on atomic coordinates and interatomic forces obtained from molecular dynamics calculations. Using these effective force constants, the phonon spectrum was calculated by the lattice dynamics method.

**Figure 13:**
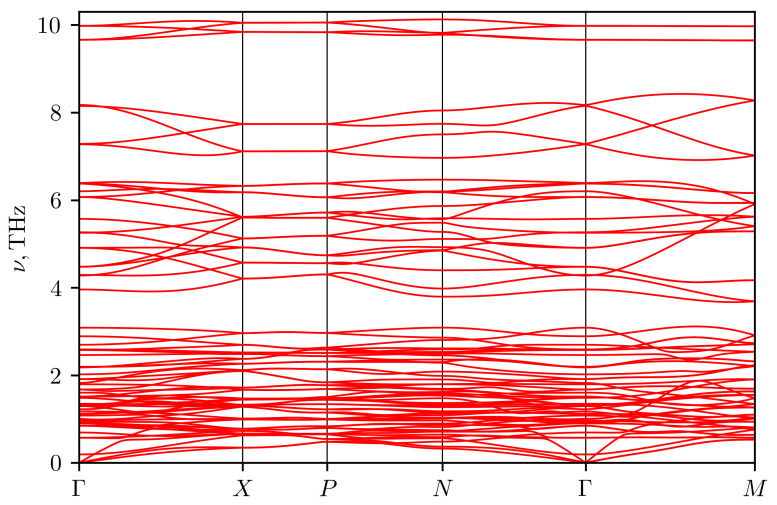
Phonon spectrum of Ag${}_{3}$CuS${}_{2}$ (I4${}_{1}$/amd) calculated at room temperature.

**Figure 14:**
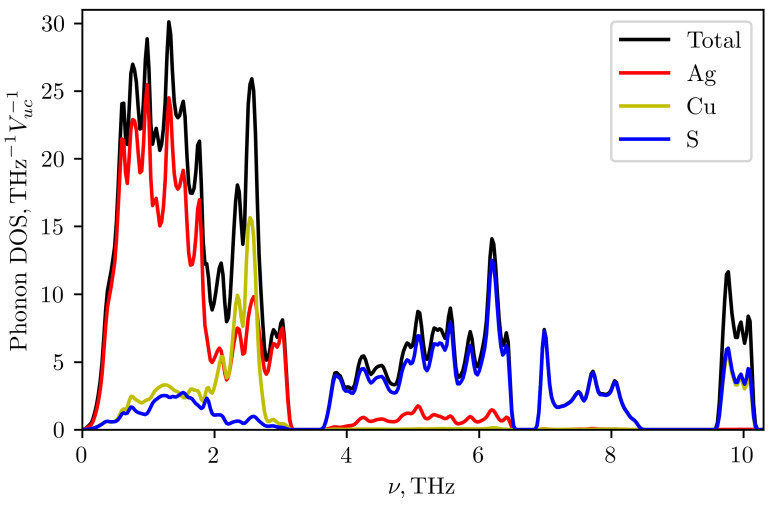
Total and atom-projected phonon density of states in Ag${}_{3}$CuS${}_{2}$ (I4${}_{1}$/amd) calculated at room temperature.

Molecular dynamics calculations were performed using the VASP program in the NVT ensemble. The calculation was carried out at the experimental values of the lattice parameters for 300 K from [11] for a supercell of 96 atoms. The cutoff energy was 400 eV, and the Monkhorst-Pack grid in the Brillouin zone of the supercell was 2×2×2. The step of integrating the equations of motion was equal to 1fs. The system was equilibrated for 1000 steps, and then data were collected from 2000 simulation steps. With the obtained data, the force constants of the 2nd and 3rd orders, which best describe the surface of constant energy, were calculated in the TDEP [29,30] program. The resulting phonon spectrum in Ag${}_{3}$CuS${}_{2}$ (I4${}_{1}$/amd) is depicted in the Figure 13. It demonstrates the absence of imaginary modes, i.e., the stabilization of the structure. The Figure 14 shows the phonon density of states and the contribution to the latter from vibrations of atoms of each type. Acoustic modes occupy a small frequency range from 0 to 1 THz. From 0 to 2 THz, the main contribution to vibrations comes from silver atoms. In the range from 2 to 3 THz, there is a narrow peak associated with copper ions vibrations with an additional contribution from silver ions. Then, there are three bands of optical phonons. The frequency range from 3.5 to 6.5 THz is associated mainly with vibrations of sulfur atoms with a small addition from vibrations of silver atoms. Modes in the range 6.5–9 THz are only of sulfur atoms, and a narrow band near 10 THz contains approximately equal contributions from vibrations of sulfur and copper.

**Figure 15:**
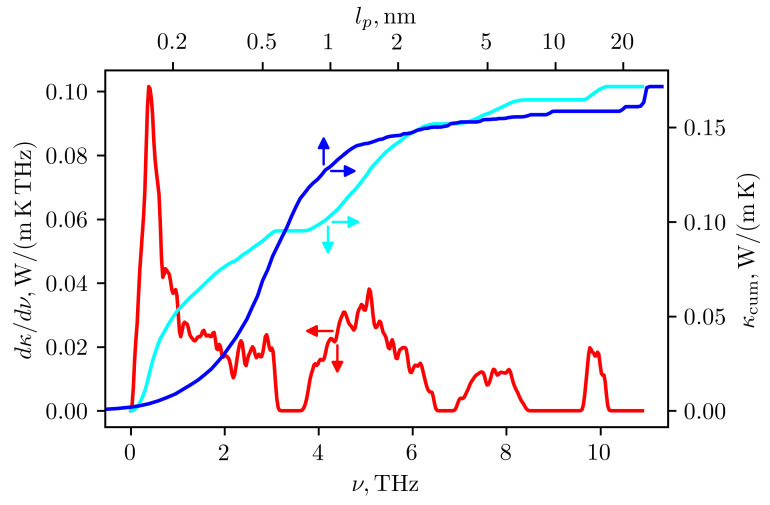
Spectral (red curve) and cumulative (cyan curve) contributions of phonons into lattice thermal conductivity at 300 K as a functions of phonon frequency. Blue curve shows cumulative thermal conductivity as a function of phonon mean free path $l_{p}$. Arrows are directed to the axes used for plotting corresponding curves.

The lattice thermal conductivity of Ag${}_{3}$CuS${}_{2}$ at room temperature was calculated using the TDEP program with the obtained force constants of the 2nd and 3rd order. Due to the large number of optical modes with low group velocity and the strong anharmonicity of vibrations, the calculation gave very low thermal conductivity values: 0.14 and 0.2 W/(m K) in the a-b plane and along the c axis, respectively. The Figure 15 shows the frequency-resolved phonon contributions to the thermal conductivity averaged over directions (red curve). In addition, the cumulative contribution to thermal conductivity from phonons with different frequencies are also shown (cyan curve). It can be seen that approximately half of the contribution comes from acoustic and low-frequency optical modes with frequencies up to 3 THz, where the contributions of silver and copper vibrations are predominant. Almost the entire remaining contribution comes from optical modes associated with vibrations of sulfur atoms in the range from 3.5 to 6.5 THz. Experimental data also give a low value for thermal conductivity—about 0.5 W/(m K), but it is noticeably higher than the calculated value. The reason for the discrepancy between the estimates and experiment requires further investigation. One possible reason for the discrepancy may lie in the description of the phonon spectrum. Experimental data on the phonon frequencies of jalpaite are not available in the literature, but from a comparison of the Figure 12 and Figure 13 it can be seen that the spectrum obtained by the TDEP method is shifted to lower frequencies: the frequency range has decreased from 12 to 10 THz. The difference in the the range of phonon frequencies can be due to different approximations for density functional used in [28] (PBEsol) and in the present work (PBE). In addition, an account of anharmonicity by effective harmonic potential, obtained in TDEP approach, can result not only in the stabilization of phonon modes but also in a decrease of maximum frequencies in the spectrum depending on the type and sign of anharmonicity (see, e.g., [29]). In addition, the cumulative contribution to thermal conductivity from the phonons with different mean free paths $l_{p}$ showed that up to 80% of thermal conductivity is due to phonons with $l_{p}$ < 1.5 nm (see blue curve in the Figure 15). For such small mean free paths the usual approach based on the kinetic equation for the description of thermal conductivity may becomes questionable. It would be interesting in the future to compare the obtained spectrum with the results of a direct calculation of the quasiparticle spectrum of phonons using data on molecular dynamic trajectories [31]. The comparison of thermal conductivity estimates with direct calculations using the molecular dynamics would also be desirable.

## 6. Conclusions

In this work we presented experimental and theoretical study of band structure and thermelectric properties of jalpaite (Ag${}_{3}$CuS${}_{2}$). The Seebeck coefficient, electrical conductivity and thermal conductivity were measured in a broad temperature range. The samples have intrinsic conduction with high resistivity above 1 kΩcm at room temperature, that decreases down to 0.1 Ωcm at 600 K. The temperature dependence of resistivity demonstrate several peculiarities close to the phase transition temperatures given in [11]. The Seebeck coefficient is negative and has a value down to −1000 μV/K near room temperature and around −400 μV/K at 600 K. Close to room temperature and below the Seebeck coefficient has a tendency of a sign change that can be caused by approaching the low-temperature phase transition. The thermal conductivity demonstrates unusual temperature dependence and the value of 0.5 W/(m K) at the room temperature. Low values of thermal conductivity and high Seebeck coefficient can be preferential for thermoelectric applications under proper doping conditions.

Theoretically, electronic band structure, Seebeck and Hall coefficients were studied at the room temperature. The calculations showed that the material tends to demonstrate negative Seebeck coefficient in intrinsic doping region with the magnitude close to experimental values. The investigation of point defects using supercell approach showed that the copper and silver vacancies can lead to the p-type doping, while sulfur vacancies should lead only to decrease of hole mobility. The anaharmonisity stabilised phonon spectrum at room temperatures was obtained using temperature dependent effective potential approach. Both, the theoretical estimates and the experiment, yield a low lattice thermal conductivity due to complex crystal structure.

## Figures and Tables

**Table 1:** Atomic positions in Ag${}_{3}$CuS${}_{2}$ (I4${}_{1}$/amd).

Trots et al. [11] (Expt.)				
Ag1	8c	0	0	0
Ag2	16g	−0.3127	−0.0627	0.875
Cu	8e	0	0.25	0.5319
S	16h	0	−0.0023	0.2146
**PBE (This Work)**				
Ag1	8c	0	0	0
Ag2	16g	−0.3134	−0.0634	0.875
Cu	8e	0	0.25	0.5281
S	16h	0	0.0052	0.2137

## Data Availability

The data that support the findings of this study are available from the corresponding author upon reasonable request.
